# Re-directed T cells for the treatment of fibroblast activation protein (FAP)-positive malignant pleural mesothelioma (FAPME-1)

**DOI:** 10.1186/1471-2407-12-615

**Published:** 2012-12-22

**Authors:** Ulf Petrausch, Petra C Schuberth, Christian Hagedorn, Alex Soltermann, Sandra Tomaszek, Rolf Stahel, Walter Weder, Christoph Renner

**Affiliations:** 1 Department of Immunology, University Hospital Zurich, Rämistr. 100, 8091, Zürich, Switzerland; 2 Department of Oncology, University Hospital Zurich, Rämistr. 100, 8091, Zürich, Switzerland; 3 Institute of Surgical Pathology, University Hospital Zurich, Schmelzbergstr. 12, 8091, Zurich, Switzerland; 4 Division of Thoracic Surgery, University Hospital Zurich, Rämistr. 100, 8091, Zurich, Switzerland

## Abstract

**Background:**

Asbestos is the main cause of MPM in industrialized countries. Even since asbestos is banned in most developed countries, the peak wave of MPM incidence is anticipated for the next years due to the long latency of asbestos induced MPM. MPM patients not eligible for surgical procedures like decortication or pleuro-pneumectomie have a median survival of 12 months with palliative chemotherapy. Therefore, new therapeutic approaches are of crucial need in this clinical situation.

**Methods/design:**

This is a phase I trial for patients with malignant pleural mesothelioma with pleural effusion testing the safety of a fixed single dose of 1x10^6^ adoptively transferred FAP-specific re-directed T cells given directly in the pleural effusion. Lymphocytes will be taken 21 days before transfer from peripheral blood. CD8 positive T cells will be isolated and re-programmed by retroviral transfer of a chimeric antigen receptor recognizing FAP which serves as target structure in MPM. At day 0 of the protocol, re-directed T cells will be injected in the pleural effusion and patients will be monitored for 48h under intermediate care conditions. AE, SAE, SADR and SUSAR will be monitored for 35 days and evaluated by an independent safety board to define any dose limiting toxicity (DLT). No further patient can be treated before the previous patient passed day 14 after T cell transfer. The protocol will be judged as save when no DLT occurred in the first 3 patients, or 1 DLT in 6 patients. Secondary objectives are feasibility and immune monitoring.

**Discussion:**

Adoptive T cell transfer is a new and rapidly expanding branch of immunotherapies focusing on cancer treatment. Recently, objective responses could be observed in patients with chronic lymphatic leukemia treated with adoptively transferred CD19-specific re-directed T cells. The choice of the target antigen determines the possible on-target off-tissue toxicity of such approaches. There are reports of severe toxicity in patients who received T cells intravenously due to unexpected expression of the target antigen (on-target) in other tissues than the tumor (off-tissue). To minimize the risk of on-target off-tissue toxicity and to maximize the on-target anti-tumor effect we propose a clinical protocol with loco-regional administration of re-directed T cells. FAP-specific T cells will be directly injected in the pleural effusion of patients with MPM.

**Trial registration:**

ClinicalTrials.gov (NCT01722149)

## Background

### Malignant pleural mesothelioma

The worldwide incidence of malignant pleural mesothelioma (MPM) is still increasing due to the use of asbestos with regional differences ranging from 7 per million (Japan) to 40 per million (Australia) inhabitants per year [1]. In Europe, the annual incidence is about 20 per million inhabitants. Even since asbestos is banned in the industrialized world, it is still widely utilized in developing countries. Therefore, a wave of new cases is predicted in developing countries, for example in India [2].

Even when MPM is diagnosed at limited stage and in patients with good performance status, MPM is an incurable cancer despite the sequential use of different treatment modalities. Neither surgery nor radiotherapy alone has resulted in increased survival [3]. The survival of treated patients has a median of less than 12 months after diagnosis. Recently, the median survival could be improved by the sequential use of neo-adjuvant chemotherapy, extensive thoracic surgery including pleuro-pneumectomie and adjuvant radiation therapy [4-6]. However, the median survival is still not exceeding more than 2 years after diagnosis. Since the multi-modal approach is not evaluated prospectively so far, the impact of surgery on MPM is still debated [7]. Ultimately, there are attempts to prospectively test the role of surgery [8]. The European Organization for Research and Treatment of Cancer (EORTC) performed a retrospective analysis to identify prognostic factors. Important factors in MPM patients were histological subtype, performance status, and disease stage at time of diagnosis [9].

### Immunotherapy

**Figure 1:**
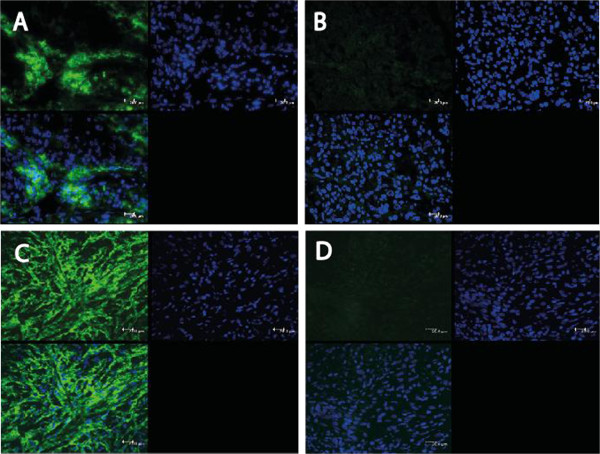
**For the evaluation of the expression of FAP in MPM immunofluorescence (IF) microscopy was performed with the F19 antibody (A, C) or an irrelevant anti-CD20 antibody only (B, D).** Epithelioid (**A**, **B**) and sarcomatoid (**C**, **D**) MPM were analyzed. FAP staining is depicted in green. Cell nuclei were stained with DAPI (blue).

Due to the unacceptable poor prognosis of MPM patients, new therapeutic approaches have to be developed. Immunotherapy is an attractive approach to add to current therapeutic concepts. Adoptive T cell transfer as part of immunotherapy is already used in clinical trials [10]. The main focus so far has been on the treatment of malignant melanoma due to the fact that melanoma antigens are known to be specific for the respective tumor cells. These so-called tumor-associated antigens (TAA) are the target structure to guide adoptively transferred T cells to the tumor. Over the last years, more and more TAAs became known in other cancer types [11,12]. However, the tumor consists not only of cancer cells but also of tumor stroma which includes fibroblasts, blood vessels, lymph vessels, and extracellular matrix proteins [13]. The tumor stroma itself appears to be a potential target for immunotherapy due to the over-expression of certain proteins that can act as target structures. Fibroblast activation protein (FAP) is such a target structure expressed by activated fibroblast in about 90% of all epithelial cancers [14]. We could identify FAP expression in all MPM subtypes (Figure 1). However, one major road block for the induction of TAA-specific T cells is the restricted T cell repertoire. Since the majority of tumor-antigens are self-antigens, no reactive T cell clones are present to recognize the cancer cells as they are deleted in the thymus [15].

### Re-directed T cells

**Figure 2:**
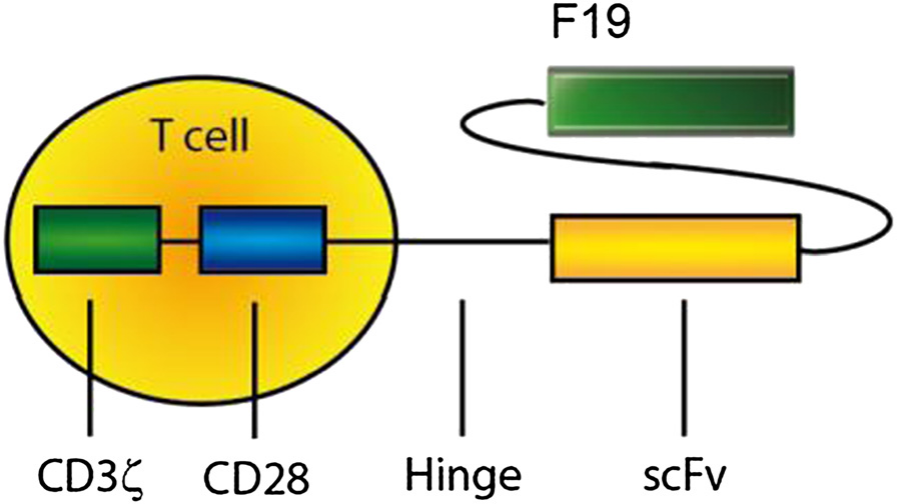
**The chimeric antigen receptor F19 is a fusion protein which consists of four functional domains.** The scFv F19 serves as binding domain and binds to FAP. The IgG Fc-domain functions as a spacer (hinge); the CD28 signalling domain leads to co-stimulation and the CD3ζ signalling domain activates the T cell if the receptor is cross-linked via antigen binding. The CAR will be expressed in CD8 positive T cells with unknown endogenous specificity.

The generation of re-directed T cells may overcome this road block of a restricted T cell repertoire as shown by objective responses in patients with chronic lymphatic leukemia treated with CD19-specific re-directed T cells [16]. Re-directed T cells harbor a chimeric antigen receptor (CAR) that allows for the recognition of tumor-antigens and, therefore, leads to the activation of T cells independently of their original specificity (Figure 2) [17]. Recently, we reported the *in vitro* and *in vivo* activity of re-directed T cells targeting NY-ESO-1 positive and HLA2 positive tumors [18]. In parallel, we developed fibroblast activation protein (FAP)-specific re-directed T cells containing an immunoreceptor coding for the scFv recognizing (F19) linked to an IgG-spacer-domain, the CD28 co-stimulatory domain and the CD3ζ chain (Figure 2). Re-directed FAP-specific CD8+ T cells showed antigen-specific functionality in vitro and were protective in a xengraft model (manuscript under review). T cells will be re-directed by gene transfer *ex vivo* under GMP conditions. The CAR will be transferred into the T cells by retroviral transduction using the pBullet vector system that has already proven its safe use with re-directed T cells in clinical trials [19]. The preparation of the transgene-containing retrovirus supernatant met the quality and safety control criteria [20]. There were no descriptions of any secondary malignancy. However, recently an immune response against the vector system could be detected [21]. To target FAP, we will use the single-chain fragment of the humanised F19 antibody. The murine F19 antibody and a humanized version (sibrotuzumab) have already been used in two phase I trials with 17 patients [22] and 26 patients [23], respectively, and one phase II trial with 25 patients [24]. The biodistribution profile of the radio-labelled antibody in the phase I trial showed specific accumulation in FAP-positive hepatic metastasis of patients with colorectal cancer. No toxicity was observed in this trial [22]. The other trial was designed to identify the maximum tolerated dose. 218 doses were given and only one episode of dose-limiting toxicity was observed. The maximal tolerated dose was not reached. The one dose-limiting toxicity was back pain [23]. In the other phase II trial, 5 patients showed drug toxicity including rigors/chills, nausea, flushing and one incidence of bronchospasm [24].

## Methods/design

### General study design

**Figure 3:**
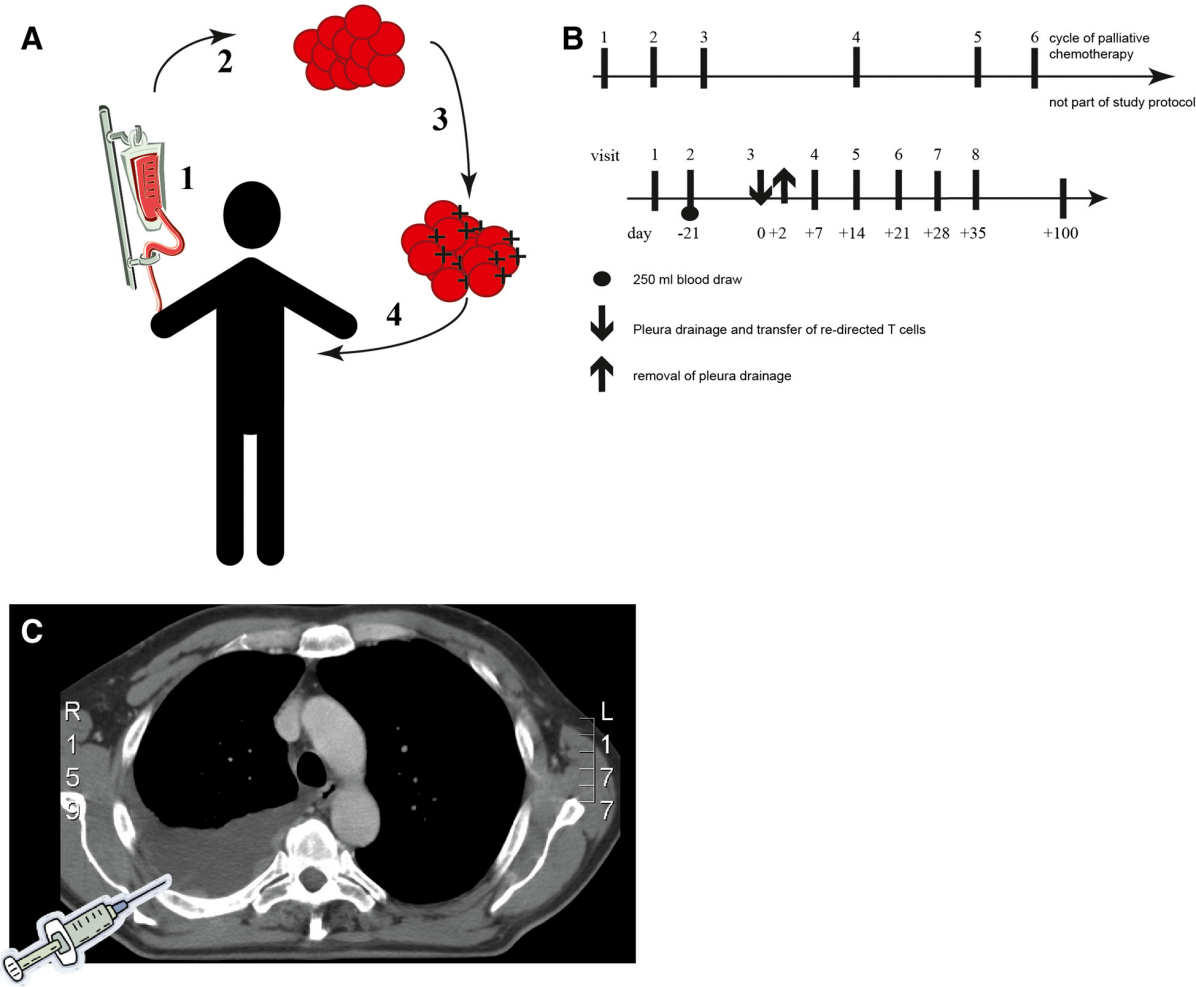
**(A) Adoptive transfer of re-directed T cells: (1) 250 ml blood withdrawal at day −21, (2) separation of CD8+ T cells, (3) retroviral transduction of the CAR (+) specific for FAP, (4) adoptive transfer of re-directed T cells day 0. **(**B**) Study course: As concomitant palliative therapy chemotherapy with cycles of 21 days will be given before and after adoptive transfer. Re-directed T cells will be given 14 days after third cycle of palliative chemotherapy into the pleural effusion. Blood draw of 250 ml blood will be performed at day −21 for the production or re-directed T cells. Patients will be continuously monitored at an intermediate care unit (ICU) for the first 48h after the adoptive transfer of re-directed T cells into the pleural effusion (day 0). 30 min before the application of the re-directed T cells 16 mg Dexamethason will be given i.v. as pre-medication. The fourth cycle of palliative chemotherapy will be started 14 days after transfer. Palliative chemotherapy is not part of the study protocol. Radiation therapy is not permitted. Pleurodesis is not allowed until completion of the study. Supportive or other palliative measures are permitted without any exceptions. (**C**) CT scan of the thorax of a patient with MPM. The tumor nodules are attached to the pleural causing pleural effusion. The schematic syringe represents the application of the re-directed T cells in the pleural infusion resulting in a proximity of re-directed T cells and tumor.

This is a phase I trial for patients with malignant pleural mesothelioma. A fixed single dose of adoptively transferred FAP-specific re-directed T cells will be given in the pleural effusion (Figure 3). Patients are eligible for the trial based on the inclusion and exclusion criteria presented in Tables 1 and 2.

**Table 1:** Inclusion criteria

**Patients fulfilling all of the following inclusion criteria may be enrolled in the study**	
Inclusion criteria:	
· Histologically or cytologically confirmed and documented malignant pleural mesothelioma with pleural effusion,	
· Male and Female patients 18 years to 75 years of age,	
· Signed Informed Consent after being informed,	
· Patients medically and/or functionally at screening not accessible for surgical treatment with planned third cycle of palliative chemotherapy in 21 days,	
· Bone marrow function: hemoglobin ≥ 100 g/L; white blood cell count (WBC) ≥ 3.0 x 10^9^/L; absolute neutrophile count (ANC) ≥ 1.5 x 10^9^/L; platelet count ≥ 100 x 10^9^/L,	
· Hepatic: aspartate transaminase (AST) and alanine transaminase (ALT) ≤ 2.5 times upper limit of normal (ULN)); bilirubin ≤ 1.5 x ULN,	
· Renal: creatinine ≤ 2 mg/dL and creatinine clearance ≥ 45 mL/min,	
· No concomitant treatment with systemic corticosteroids, or any other immunosuppressive agents,	
· The patient has received no major organ allograft,	
· HIV-negative,	
· HBV and HCV negative,	
· No uncontrolled bleeding disorder,	
· Patients of child-producing potential must agree to use contraception while enrolled in the study and for 24 months after the adoptive transfer.	

**Table 2:** Exclusion criteria

**The presence of any one of the following exclusion criteria will lead to exclusion of the subject:**	
Exclusion criteria:	
· Contra-indications to the class of TpP, e.g. known hypersensitivity or allergy to the investigational product,	
· Contra-indications on ethical grounds,	
· Women who are pregnant or breast feeding,	
· Intention to become pregnant during the course of the study,	
· Lack of safe contraception:	
Safe contraception is defined as follows:	
Female and male subjects of childbearing potential, using and willing to continue using a medically reliable method of double barrier contraception for the entire study duration and the next 2 years, such as oral, injectable, or implantable contraceptives, or intrauterine contraceptive devices in combination with preservatives. Or subjects who are using any other method considered sufficiently reliable by the investigator in individual cases.	
Please note that subjects who are surgically sterilized/hysterectomized or post-menopausal for longer than 2 years are not considered as being of child bearing potential.	
· Known or suspected non-compliance, drug or alcohol abuse,	
· Patients with medical history of coronary heart disease (CHD), stroke or peripheral vascular disease (PVD),	
· Patients with medical history of autoimmune disease such as multiple sclerosis, lupus, rheumatoid arthritis, inflammatory bowel disease or small vessel vasculitis,	
· Regular intake of immune-modulating drugs,	
· Inability to follow the procedures of the study, e.g. due to language problems, psychological disorders, dementia or confusional state of the subject,	
· Participation in another study with investigational drug within the 30 days preceding and during the present study,	
· Previous enrolment into the current study,	
· Enrolment of the investigator, his/her family members, employees and other dependent persons.	

Three patients who are at the time point of screening not operable will be treated with re-directed T cells administered into the pleural effusion after completion of 3 cycles of palliative chemotherapy (Figure 3). In the case of one AE grade III/IV or one SAE - both judged to be treatment related by an independent safety monitoring board - the patient number will be expanded to 6 patients. The study will be stopped if one additional DLT occurs also judged to be treatment related.

Patients will be treated with 1x10^6^ re-directed FAP-specific T cells injected in the pleural effusion. The study ends 35 days after adoptive T cell transfer. Re-directed FAP-specific T cells will be administered at day 0 (day 14 of the third cycle of palliative chemotherapy). The study is designed to demonstrate safety of 1x10^6^ re-directed FAP-specific T cells. The next patient will be enrolled earliest, when the previous patient completed day +14 and the safety monitoring board has not declared any DLTs. The palliative chemotherapy is not part of the study protocol. Due to nature of the study only descriptive statistics will be applied. Means, medians, standard deviations and ranges will be provided for continuous outcomes, and frequencies will be reported for categorical data. The study is approved by the local ethical committee (Kantonale Ethikkommission) of Zurich (KEK-ZH-Nr. 2012/0106), listed at ClinicalTrials.gov (NCT01722149) and in accordance with the Declaration of Helsinki.

### Study objectives

#### Primary objective

The primary objective is to test safety of a fixed single dose of adoptively transferred FAP-specific re-directed T cells in the pleural effusion. Therefore, all grade III/IV AEs and SAEs will be measured. For the safety assessment, grade III/IV AEs and SAEs will be collected until day +35. The primary safety variables are abnormalities that are judged to be treatment-related and dose limiting toxicity (DLT) by an independent safety monitoring board after the patient passed day +35. Incidence and severity of treatment-related laboratory abnormalities, graded according to National Cancer Institute Common Terminology Criteria for Adverse Events (NCI-CTCAE) version v4.03 criteria as grade III-IV. The protocol will be judged as save when no DLT occurred in the first 3 patients, or not more than 1 DLT in 6 patients.

### Secondary objectives

#### Feasibility

Number of patients who received re-directed T cells at day 0 of whom 250 ml of blood were taken at day −21. The study protocol will be declared as feasible when all patients from whom blood was taken, have received transduced T cells.

#### Immune monitoring

Quality of generated re-directed T cells will be tested on day 0. Therefore, frequency assessment of subpopulations of re-directed T cells and assessment of transduction efficacy using flow cytometry will be performed using flow cytometry (monoclonal antibodies: human anti-CD3, human anti-CD4, human anti-CD8, human anti-CD56, human anti-CD28, human anti-CD27, human anti-CD45RA, human anti-CCR7, human anti-IgG). Antigen-specific IFNγ-release after stimulation with FAP expressing cells will be used to test for T cell function. Furthermore, cytotoxicity assays will be performed to determine antigen-specific cell killing after stimulation with FAP expressing cells. The cytokine composition of pleural effusion will be measured at days 0 and +2 by ELISA for IL-2, IL-4, IL-10, TGF-β, TNF-α and IFNγ to assess the local microenvironment. In parallel, the cytokine composition of peripheral serum will be analysed on days −21, 0, +1, +2 and +7 by ELISA for IL-2, IL-4, IL-10, TGF-β, TNF-α and IFNγ. The number of re-directed T cells will be measured in the peripheral blood on days −21, 0, +1, +2, +7 by flow cytometry (human anti-IgG mAB and human anti-CD8 mAB) to assess for lymphocyte migration out of the pleural cavity.

### Dose rationale

This study is designed to show safety of a single dose. Therefore, we do not plan for a dose escalation. We decided to take the lowest dose of re-directed T cell recommended for second generation (CD28 signaling domain) CARs targeting a so far not tested tumor related antigen (FAP) [25]. We will administer 1x 10^6^ re-directed T cells into the pleural effusion.

## Discussion

First-line chemotherapy is the standard treatment option for patients not assessable for surgery. Since palliative first-line chemotherapy results in a median overall survival of 12 months, new therapeutic approaches have to be developed. Therefore, we proposed a concept of loco-regional adoptive transfer of re-directed T cells. We demonstrated target expression of FAP in all subtypes of MPM. Thus, FAP-specific re-directed T cells can be employed in the proposed clinical protocol. The clinical data, so far, using F19 (murine anti-FAP mAB) or sibrotuzumab (humanized F19) showed antibody accumulation in the tumor tissue indicating target specificity [22]. However, the clinical response to both antibody forms was marginal [23]. In contrast to antibodies, re-directed FAP-specific T cells will make use of the specificity of a humanised F19 antibody and combine it with the cytotoxic machinery of CD8 positive effector T cells [26]. Therefore, we expect increased clinical responses [27]. However, the here presented protocol is not intended to proof efficacy.

The main advantage of the loco-regional application of re-directed T cells is the avoidance of immediate systemic side-effects, since the re-directed T cells will not be systemically distributed by blood flow as if administered intravenously. Intravenous application can lead to an interaction of FAP re-directed T cells with off-target tissue leading to immunological mediated toxicity [25]. On-target off-tissue toxicity has to be addressed as first goal. Due to the fact that FAP is expressed in chronically inflamed tissue [28,29], off-target toxicity has to be taken into consideration. Furthermore, FAP expression could be found in fibroatheromata [30]. Therefore, patients with a medical history of autoimmune diseases and/or coronary heart disease, stroke and peripheral vascular disease are excluded from the study. More related to the systemic delivery of T cells, massive cytokine release could be caused by adoptively transferred T cells. There is one report describing an adverse event resulting in the death of a patient after the adoptive transfer of ERBB2 specific re-directed T cells [31]. The cause of death could be partially explained by a cytokine storm. The here presented study is differently designed. Re-directed T cells will not be given intravenously; therefore, a rapid and uncontrolled systemic contact to FAP positive tissue is not likely [32]. The patients are not lympho-depleted by palliative chemotherapy; therefore, clonal competition for cytokines can still take place and lowers the activation of transferred re-directed T cells [33]. Nevertheless, re-directed T cells may expand and migrate outside the pleural effusion and cause in the acute phase anaphylaxis and ARDS within the first 48h. Later on, chronic inflammation can be induced at different sites. Since this trial is the first trial evaluating FAP-specific re-directed T cells in the pleural effusion, all enrolled patients will be treated with the lowest suggested number of cells [25] and monitored for the first 48h at an intermediate care unit.

With the herein presented trial we propose a clinical trial design to test the safety of pre-clinically evaluated FAP specific re-directed T cells for loco-regional intrapleural therapy in patients with MPM.

## Abbreviations

AE: Adverse Event; ARDS: Acute Respiratory Distress Syndrome; CAR: Chimeric antigen receptor; CD: Cluster of Differentiation; DLT: Dose limiting toxicity (DLT); ELISA: Enzyme Linked Immunoabsorbent Assay; ECOG: Eastern Cooperative Oncology Group Performance Status; FAP: Fibroblast activation protein; IFN: Interferon; IL: Interleukin; ICU: Intermediate Care Unit; mAB: monoclonal Antibody; MPM: Malignant pleural mesothelioma; SADR: Suspected adverse drug reaction; SAE: Serious Adverse Event; SUSAR: Suspected Unexpected Serious Adverse Reaction; TAA: Tumor associated antigen; TGF: Tumor growth factor; TNF: Tumor necrosis factor; TpP: Transplantation product.

## Competing interests

The authors declare that they have no competing interests.

## Authors’ contributions

UP, CR and RS designed the study. UP coordinates the trial. PCS, CH performed pre-clinical experimentation. AS is the reference pathologist for confirmation of histological diagnosis. ST is involved in sample collection and patient recruitment. CR and WW are the principle investigators. All authors have read and approved the final manuscript.

## Pre-publication history

The pre-publication history for this paper can be accessed here:

http://www.biomedcentral.com/1471-2407/12/615/prepub
